# Risk Assessment to Underpin Food Regulatory Decisions: An Example of Public Health Nutritional Epidemiology 

**DOI:** 10.3390/nu3010164

**Published:** 2011-01-20

**Authors:** Janis Baines, Judy Cunningham, Christel Leemhuis, Tracy Hambridge, Dorothy Mackerras

**Affiliations:** Food Standards Australia New Zealand, PO Box 7186, Canberra BC, ACT 2610, Australia; Email: janis.baines@foodstandards.gov.au (J.B.); judy.cunningham@foodstandards.gov.au (J.C.); christel.leemhuis@foodstandards.gov.au (C.L.); tracy.hambridge@foodstandards.gov.au (T.H.)

**Keywords:** risk analysis, food regulation, fortification, additives

## Abstract

The approach used by food regulation agencies to examine the literature and forecast the impact of possible food regulations has many similar features to the approach used in nutritional epidemiological research. We outline the Risk Analysis Framework described by FAO/WHO, in which there is formal progression from identification of the nutrient or food chemical of interest, through to describing its effect on health and then assessing whether there is a risk to the population based on dietary exposure estimates. We then discuss some important considerations for the dietary modeling component of the Framework, including several methodological issues that also exist in research nutritional epidemiology. Finally, we give several case studies that illustrate how the different methodological components are used together to inform decisions about how to manage the regulatory problem.

## 1. Introduction

Epidemiology is defined as “the study of the distribution and determinants of disease frequency in human populations” [1]. Kaldor commented on the division between two epidemiological tribes which he called “research epidemiology” (what researchers do) and “public health epidemiology” (what health departments do) [2]. He noted that randomized controlled trials, cohort studies and case-control studies were prominent designs used in research epidemiology whereas cross-sectional studies, including administrative databases such as mortality registrations, were the primary design used in public health epidemiology [2]. Another epidemiological dichotomy is “analytical epidemiology” and “descriptive epidemiology” [1]. Together, these two dichotomies imply that public health epidemiology is descriptive in approach with minimal analysis but this is not necessarily true. The real difference between the “research” and “public health” epidemiology is the focus of the question being asked. 

Nutritional epidemiology is commonly defined as “the study of the nutritional determinants of the distribution of disease” [3]. Textbooks about nutritional epidemiology tend to focus on research nutritional epidemiology and its associated methods, such as comparison of food frequency questionnaires with 24-h recalls [3,4]. They tend to overlook the use of epidemiology in food-related public health [4]. 

Food regulation agencies use both aspects of nutritional epidemiology to assess food-related risk. The literature on health risks associated with food constituents is assessed and then population dietary exposure estimates are generated from food consumption data. In this paper, we outline the Framework that join these two activities then focus in more detail on some features of dietary exposure estimation and interpretation. Finally we give some case studies that illustrate the use of different analytical approaches to answer different questions. Although this paper is based on our experience and practice at Food Standards Australia New Zealand (FSANZ) [5,6], the general approach is used by other food regulatory entities with equivalent capacity. 

## 2. Overview of the Food Regulation Context in Which Epidemiological Data are Used

Nutrients are only one of the food chemicals that interest a food regulatory agency. Others are bioactives, agricultural and veterinary residues, naturally occurring toxicants, additives, contaminants, adulterants and packaging migrants. Table 1 shows some food chemicals of international regulatory interest in recent years. Although the problem might be identified in a single country, other countries need to check whether similar problems have arisen domestically or whether they have imported affected products from the index country. 

In this paper we use “food regulation agency” as though one agency has the remit for all activities. However, the range of responsibilities described might be divided among several departments or agencies depending on the structure of the government organizations and their responsibilities. 

### The Risk Analysis Framework

The fundamental purpose of food regulation drives the use of epidemiological and other data: namely that a decision must be made either to change the regulation governing how much of a specific food chemical is permitted or to maintain the current situation (status quo). The status quo can range from no regulation for the chemical, to permission for specific concentrations in certain foods to prohibition of the chemical in the food supply. 

**Table 1:** Some food chemicals of recent international interest to food regulation agencies.

Type	Example of Food Chemical	Source and Effect	References
Adulterants (prohibited substances)	Melamine	Deliberate adulteration; renal failure and death in infants fed adulterated infant formula	[7]
Nutrients	Fluoride	Found naturally. Also added to water and toothpaste. Potential exposure to high levels following the Icelandic volcano	[8]
	Iodine	Very high levels in a soy drink due to use of a seaweed concentrate lead to hospital admissions in adults and breastfeeding neonates; the product was recalled in several countries	[9]
Bioactives	Caffeine	Found in coffee and lesser amounts in tea & chocolate. Added to some energy drinks; new research suggesting pregnant women with higher intakes were more likely to have a low birth weight infant	[10]
	Lutein	Marigold petals and some other foods; alleged to improve eye health and therefore possibly desirable to add to infant formula	[11]
Substances formed during cooking	Acrylamide	Formed during high temperature cooking, such as roasting or frying, from protein and sugars present in food; a carcinogen	[12]
	Polycyclic aromatic hydrocarbons (PAH)	Found naturally and also produced by industrial processes and by some cooking methods (e.g., barbequing, smoking). A number of PAH are known or suspected carcinogens	[13]
Additives	Certain artificial colors	Used in various foods; alleged to cause behavior problems in children	[14]
	Benzene	In the presence of acid, sodium benzoate (a preservative) can break down to benzene, a carcinogen. Benzene from this source was detected in carbonated beverages in several countries	[15]
Packaging migrants	Bisphenol A	Monomer found in polycarbonate plastics and epoxy resins used to line cans, variable effects on hormonal activity in laboratory animals	[16]
Environmental contaminants	Perchlorates	Found naturally and also man-made (e.g., in rocket fuel), in high doses, it interferes with uptake of iodine by the thyroid	[17]
	Nitrates	Found naturally in leafy vegetables but also derived from fertilizers and is used as a food additive; can be converted into nitrosamines, a carcinogen, in the body	[18]
	Dioxins	Environmental contamination from industrial sources although there are a small number of natural sources; long term exposure linked to immune system impairment	[19]
Naturally occurring toxicants	Cyanogenic glycosides	In improperly prepared cassava chips (crisps); can cause cyanide poisoning	[20]

**Figure 1:**
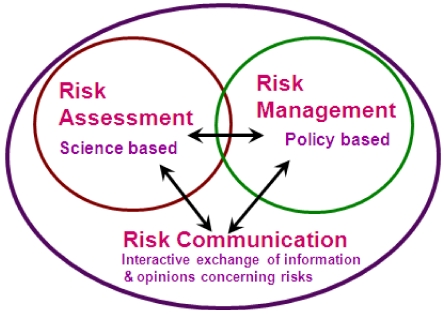
Risk analysis framework (redrawn from [21]).

The FAO/WHO Risk Analysis Framework (Figure 1) [21,22,23], is used around the world in food regulation. The Framework provides a systematic structure for assessing the risks associated with foods. It distinguishes between the description of the science (Risk Assessment) and the policy- and value-based decisions that affect the response to the problem (Risk Management). Initially, the Framework was described in generic terms with words like “exposure” rather than “intake”. More recently, it has been re-worked with a more nutritional focus which reflects that low nutrient intakes carry increased risk, and that some assessments are more easily described as benefit rather than reduction in risk [24]. 

Some of the language used in food regulation is strange to nutritionists. The term “food chemical” is used because nutrients are only one of many types of food constituent that are regulated (Table 1). In this paper, we say “dietary exposure” and food “concentration” when referring to food chemicals in general and “dietary intake” and food “composition” respectively when referring to nutrients specifically. 

**Figure 2:**
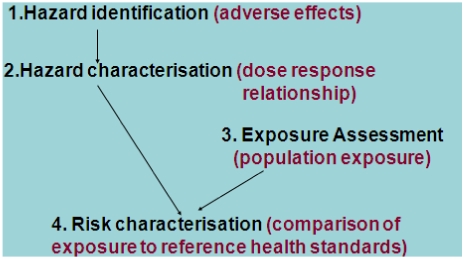
Steps in the Risk Assessment component of the Risk Analysis Framework [22,23].

Steps 1 and 2 of the Risk Assessment component of the Framework (Figure 1 and Figure 2), use the data generated from research epidemiological (and other) studies. Step 1 describes what the hazard is and the nature and severity of the health effects. Epidemiologists call hazards “exposure” and hazards (like epidemiological exposures) can be beneficial or adverse in the case of nutrients and bioactives. It is important to define the hazard clearly. For example, when assessing the risks and benefits potentially associated with fortifying the food supply with folic acid to reduce neural tube defects, it is necessary to decide whether the hazard is any form of folate or only the folic acid form because this determines what literature should be examined. Step 1 identifies whether the food chemical or nutrient is indeed a hazard and Step 2 determines the dose-response characteristics. Both research epidemiological studies and animal studies may contribute to these two steps. Reference health standards are derived from the information assessed at Steps 1 and 2, often by applying a safety factor to the lowest (adverse) effect level.

In research epidemiological studies investigating diet-disease relationships, intakes of nutrients, ideally, are generated for each study participant by applying composition data to descriptions of food consumption patterns, where consumption of specific food types has been quantified, and then summed for each participant to yield a nutrient intake per day or per week. These nutrient intakes are then used as the “exposure” or predictor variable and rates of the outcome of the disease of interest compared among people in different categories of nutrient intake. The fundamental purpose is to answer the question “is there a relationship?” It is important to rank the participants as well as possible because non-differential error in nutrient ranking can attenuate the relative risks or odds ratios severely and lead to the study finding no association even if an association truly exists. There are a number of excellent texts that cover the intricacies of exposure measurement for research epidemiological studies generally or specifically for nutritional studies [3,4,25]. For the purpose of calculating a relative risk comparing high *versus* low nutrient intakes, ranking of study participants is more important than having an accurate estimate of the absolute nutrient intake [3,4]. However, it is important to quantify absolute amounts if the results are to be used to generate advisories to the public or numerical cutpoints for regulations and reference health standards.

The promulgation/articulation of a reference health standard by a national body does not automatically indicate that a food chemical actually poses a risk in the population. The reference is a point of comparison but the population exposure must be described (Step 3) because the extent of the risk to the population depends on the prevalence of the exposure as well as the dose-response characteristics of the hazard (Figure 2). The best data for this purpose is a representative population sample survey with as much detail as possible about the type and quantity of each food consumed. This is quite different from the type of food consumption data usually available from cohort or case‑control studies. 

Exposure to food chemicals or nutrients might be from food alone or there may be other sources of exposure; for example fluoride is found in tap water and toothpaste as well as food. Water may be an important source for nutrients (iodine, iron, fluoride), contaminants (arsenic, lead) and possibly pesticide residues. Combining the information from Steps 2 and 3 yields an assessment of the proportion of the population of interest who have inappropriate exposures compared to the reference value (Step 4). Exposure in relation to the reference value, rather than the absolute exposure, is the key aspect of Step 4. 

The parallels with epidemiological work are obvious. Odds ratios or relative risk describing dose-response (Step 2) can be combined with prevalence (Step 3) to generate population attributable risk (Step 4). Various strategies which might change the prevalence of the exposure are modeled to identify how this in turn changes the population attributable risk [1]. This is conceptually equivalent to a food regulation agency projecting the change in population exposure following a proposed change to a food regulation to permit, increase use of, or restrict the amount of a food chemical in one or more foods. 

During Risk Assessment, the Risk Assessors describe the estimated dietary exposure and may forecast future possible exposures under varying conditions or different regulatory options. Risk Managers (Figure 1) then use the Risk Assessment information to examine the question “is there a problem and if so, what should be done about it?” For example, the Risk Assessors might identify that 5% of the population have dietary exposures to a food chemical greater than the reference health standard for that chemical, but it is the Risk Managers who decide whether this degree of exceedance indicates a problem that needs mitigation. In the Framework, Risk Assessment and Management are different areas of work and ideally undertaken by different groups. The Risk Managers ask the questions that the Risk Assessors answer and ongoing communication is needed between the two groups. In some countries Risk Managers and Risk Assessors are in different agencies, in others, they are in different groups in the same agency or, where staff resources are limited, they may be the same officers within an agency. The key is that the thinking and the work of each role should be undertaken separately.

For simplicity, this paper is written as though regulation is the only Risk Management option. In reality, a range of other options to mitigate risk are available to food regulators such as: voluntary industry codes of practice, guidelines or protocols, advisory statements and/or provision of educational material. Regulation imposes costs on government for enforcement and on industry for compliance. The Risk Managers need to consider the impact of these on other parts of the system such as food prices, foregone use of tax revenue for other activities and trade. Consequently, regulation is not always the preferred option for action even when a problem has been identified. Many countries now require a formal regulatory impact assessment to ensure that the option that generates the greatest net benefits is selected. The final compartment, Risk Communication (Figure 1) needs to occur throughout the process of risk assessment and risk management, keeping these two groups informed as well as interested external stakeholders.

## 3. Information Required for Dietary Modeling

Steps 3 and 4 (Figure 2) are generally conducted in tandem. The purpose of describing dietary intake of nutrients, or dietary exposure to other food chemicals, is different in food regulation from traditional research epidemiological studies. The goal is to calculate population level data such as the mean dietary exposure/nutrient intake or the proportion of the population with exposures/intakes above or below reference health standards. Consequently, the absolute measure of exposure is important, as is the mean/median and spread of the population distribution. A series of dietary calculations are done: first using current concentration data to describe current dietary exposure or nutrient intakes and then using one or more different concentrations to project the impact of possible changes to the food regulations. This iterative process is called “dietary modeling” and its fundamental purpose is to answer the question “does the regulation or proposed change in regulation result in safe dietary exposures or nutrient intakes?” [6]. 

**Figure 3:**
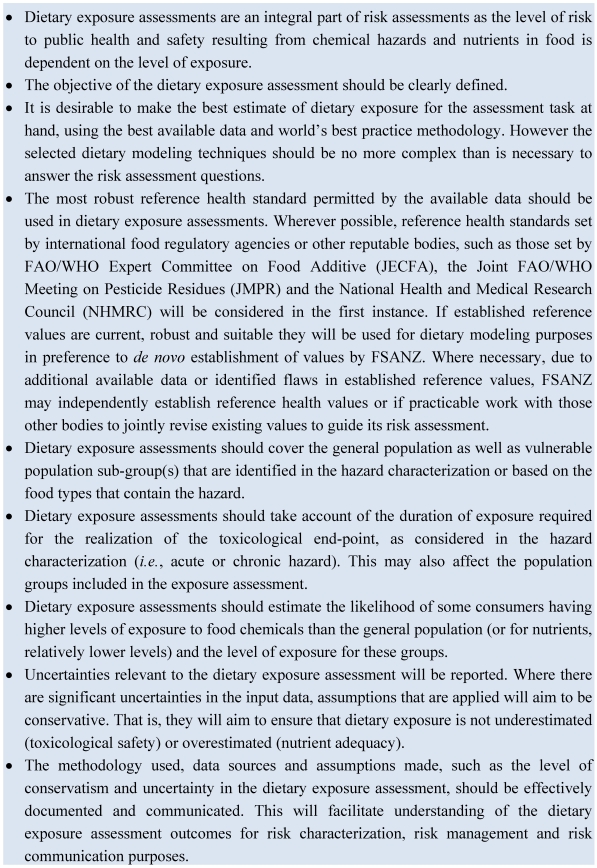
FSANZ’s best practice principles to underpin dietary modeling [6].

The required data are a set of representative food consumption data, concentration data for foods and health reference standards to interpret the population exposure/intake. During the last 20 years there have been a number of advances in the area of dietary exposure assessment for food regulation purposes [26,27]. Many decisions are required and Figure 3 summarizes FSANZ’s best practice points [6]. 

By combining the concentration of the chemical/nutrient in the food with the amount of food consumed, contributions of different foods to the total and sub-group dietary exposure or nutrient intake can be identified. The impact of possible changes in concentration of the chemical/nutrient in one of more foods can be examined. Agencies such as FSANZ have custom-designed software to manage the large datasets and perform the calculations required. There are also programs being developed internationally that would be available for anyone to use with their own data. For example, the WHO Intake Monitoring, Assessment and Planning Program (IMAPP) [28] is being developed to estimate appropriate levels of vitamins and minerals for use in food fortification. Users can load in their own data, or select from some inbuilt data for some parameters.

### 3.1. Reference Health Standards for Food Chemicals

Reference health standards derived from Steps 1 and 2 (Figure 2) are required to interpret population dietary exposures to food chemicals or nutrient intakes and characterize the risk to the population (Step 4, Figure 2). We discuss health reference health standards first. These must be examined prior to the dietary exposure/intake calculation to ensure that the concentration/composition data and calculation methods match the way the health reference standard is expressed. 

Nutrients are unusual among the food chemicals in having health reference values that are set for different population groups—by age and sex—and also for life stages such as pregnancy and lactation (Table 2). Therefore the age, sex and life stage of those reporting the nutrient intakes must be known so that intakes can calculated and interpreted against the health references. Nutrients are also unusual in having two reference health standards—one describing the risk of inadequate intake and the other risk of excessive intake. Although expressed on a per day basis, this is for convenience and these references are best applied to estimates of usual or long-term nutrient intake, *i.e.*, predicted intake over many days or weeks. 

For most food chemicals, reference health standards are expressed per kilogram body weight, not by age and sex. The average weight of the population of interest or, preferably, the body weight of each individual survey respondent, must be known to allow interpretation of dietary exposures against the reference health standards. These might be expressed per week or per month rather than per day (Table 2). For food chemicals with long term effects, it is assumed that usual long term dietary exposure has been estimated for comparison with the reference health standard. The period to achieve a steady state is approximately four times the half-life of the compound in the body [29]. The longest known half-lives are those for dioxins (11 years) [30] and cadmium (15 years) [31]. Excursions of dietary exposure over the relevant reference health standard do not necessarily indicate a long term risk when averaged over the correct length of time, even if the excursions occur over several days, weeks or months during a lifetime. 

The Acute Reference Dose (Table 2) is unusual because it is set for food chemicals, such as pesticide residues or contaminants, which might cause harm shortly after the food is consumed. Hence exposure on an occasion of eating or single 24-h dietary exposure, as appropriate, is estimated for comparison with this type of reference health standard.

The most robust and current reference health standard should be used in dietary exposure assessments. Countries might adopt values set by various FAO/WHO committees [32,33] which are established based on advice from experts from various fields, including those from national regulatory agencies. Alternatively countries might develop their own standards. This will depend on the chemical being assessed, the amount of data available or whether other agencies/committees have done a prior assessment. The Risk Assessors or the agency must to determine the standard most appropriate to the population being assessed. 

**Table 2:** Reference health standards of food chemicals for assessing human intake.

Food chemical	Focus	Terminology	Abbreviation	Basis
Nutrient	Adequacy	Average nutrient requirement	ANR *	Total daily amount with separate values by age, sex, life stage
	Excess **	Upper Level of Intake	UL	Total daily amount with separate values by age, sex, life stage
Additives	Excess	Acceptable daily intake	ADI	per kg body weight/day
Agricultural and veterinary chemical residues	Excess (chronic)	Acceptable daily intake	ADI	per kg body weight/day
	Excess (short term)	Acute Reference Dose	ARfD	per kg body weight/day
Contaminants and naturally- occurring toxicants	Excess (chronic)	Provisional tolerable daily/weekly/monthly intake	PTDI/PTWI/PTMI	per kg body weight/day, week or month
	Excess (short term)	Acute Reference Dose	ARfD	per kg body weight/day

* Also called the Estimated Average Requirement (EAR); used as the short-cut calculation instead of the Probability Approach, provided certain assumptions are met; ** Some metal nutrients are also contaminants and have PTWIs as well as ULs.

Both excessive intake and adequacy of intake are considered for nutrient risk assessments. As there is no international standardization of nutritional terminology, we use the Codex Alimentarius terms [24] for the concepts (Table 2). Comparing nutrient intake to an Upper Level of Intake (UL) is conceptually the same as comparing any other food chemical to the ADI, PTWI, *etc.* The purpose of an adequacy assessment is to estimate the proportion of the population who have inadequate nutrient intake over the long-term. Previously the proportion lying below the 98th centile of the requirement distribution (called the Recommended Dietary Allowance in some countries) was often used. When population average intake exceeds the average requirement, this overestimates the true proportion with inadequate nutrient intakes because a person with an intake just below the 98th centile on the requirement distribution has only a 2–3% chance of having an inadequate intake. Even those with nutrient intakes equal to the average requirement have only a 50% chance of having an inadequate intake, not a 100% chance [34]. To calculate the proportion of the population of interest with inadequate nutrient intakes, the probability that the intake of each individual is inadequate is calculated and summed over the whole population [35]. This is commonly referred to as the Probability Approach. Conveniently, provided certain assumptions are met, the proportion lying below the Average Nutrient Requirement (called the Estimated Average Requirement in some countries) provides a good approximation to the result that would be obtained from the Probability Approach [35,36]. This is quicker to calculate and it is more commonly used as the calculation method to estimate the proportion with inadequate nutrient intakes. 

### 3.2. Food Consumption Data

Dietary exposure estimates are an indirect measure of health owing to imperfect absorption, metabolism and excretion of food chemicals and errors in describing food consumption and measuring chemical concentrations. Direct measures or biomarkers of health status are more desirable for determining whether a health problem exists in the population. However dietary exposure estimates, and therefore food consumption data, are needed to identify which food(s) might be regulated following the identification of a health problem. They might also be used as a surrogate for health status when resources do not allow the collection of biomarker information. 

A number of different types of food consumption data might be available in a country. A national survey that collected detailed daily food consumption data from a large representative sample of all ages allows estimation of the full distribution of food consumption amounts in different population groups. Consequently it is more useful than composite data such as household budget surveys or per capita food disappearance data. Ideally, multiple days of records would be available from each individual so that the distribution of the usual dietary exposure to food chemicals can be derived, rather than dietary exposure on a single day. 

The dataset from a national survey commonly contains the foods eaten by each person and the associated nutrients. Foods are often composites of many ingredients, for example bread contains flour, yeast, salt, sugar, milk, preservatives, *etc.* Some food chemicals, such as additives, are regulated at the food level (e.g., amount of preservative in bread) and so the dataset can be used for estimating dietary exposure to these. Other chemicals, such as pesticides and contaminants, are regulated at the raw commodity level. If dietary exposure to a pesticide residue used on wheat is being estimated, then using the weight of the bread eaten would overestimate the amount of flour, and consequently pesticide residue, which was consumed. For this type of model, foods have to be disaggregated into their component ingredients and these are used in the models. Nutrition survey food grouping systems may have a nutrition focus, for example apple pastries might be classed separately from fruit or bread. When investigating pesticides used on wheat or apples it is important to capture the wheat or apple from this type of food as well as the more obvious foods made from these ingredients. Careful thought is required to ensure that all sources of a pesticide residue or contaminant are included, for example, should fish sauce be included if estimating contaminants from fish? 

Food frequency questionnaire data are less useful in this context because of the uncertainty in serving size description (if used) and the grouping of many foods together. They generally rank food consumption of individuals well, but this is not the purpose in dietary modeling for food regulation. However, they can be useful for identifying the proportion of high and low consumers of particular groups of foods in a given population. Sometimes this type of separate study might be the only source of information about a sub-group that was inadequately sampled in a national survey [37].

### 3.3. Food Chemical Concentration Data

Nutritionists would be familiar with the variability in nutrient content across different types of foods. However, food composition tables commonly publish a single value of each food type and so the variability in content across different samples within a food type is often overlooked. This variability applies to natural foods as well as processed foods and is due to season, soil, growing conditions, cultivar as well as processing factors such as different recipes between brands and random batch-to-batch variation within brands. For some food chemicals such as nutrients, it may be reasonable to assume that there is an approximately normal distribution around a mean value. For other chemicals, there may be many foods with undetectable levels owing to the food being grown in an uncontaminated area, non-use or post-harvest breakdown of pesticides, non-use of additives in manufacturing by some producers or a few foods with high levels of a contaminant due to natural variation, *etc.* In this case, the distribution of chemical concentration may be highly skewed for a particular type of food or it may have spikes at a small number of values rather than a continuous distribution. An important decision is how to treat foods with no detectable level of the chemical in the dietary exposure estimate; there are often different approaches for different chemicals [6]. For contaminants, common practice is to assign half the limit of reporting to samples with non-detect values. If this has to be applied to a large number of foods, then population exposure could be substantially overestimated. For nutrients, half the limit of reporting is used so that intakes are not over- or underestimated because both essentiality and excess are usually assessed. However, if it is certain that a chemical has not been used in particular foods, for example food additives with no permissions in particular foods, or pesticides not used on a particular food, then a non-detect value might be assigned a zero value. Recalculating the exposure estimate using different values can be carried out to determine whether the decision about how to treat the non-detects alters the estimate of exposure importantly. 

The quality of data for the relevant chemical needs to be assessed prior to a dietary modeling exercise. It might not be appropriate to combine several data sets into a single concentration dataset, particularly if foods were analyzed many years previously or were sampled and analyzed using different methods. One solution to filling in a gap for a chemical that is added to foods is to assume that the food contains the chemical at the maximum permitted level for that food. This generates a worst case scenario. If the resultant population dietary exposure estimate exceeds the reference health standard, then it would be worth commissioning food analysis or obtaining data from manufacturers to determine the true concentration of the chemical in order to refine the estimate of dietary exposure, whereas this may be deemed unnecessary if there is no exceedance. 

A further consideration is the comparability of the form of the chemical to that defined in the reference health standard. For example, in Australia, sodium and potassium nitrites are permitted forms for adding nitrite to food but the reference health standard is for nitrite and so the mass of nitrites needs to be calculated from the mass of nitrite salts used. If the reference health standard is for a group of related substances, for example vitamins A or E or dioxins, then they need to be summed, using equivalence factors if appropriate, before the dietary exposure or nutrient intake can be compared to the reference health standard. 

Total diet studies are a particular approach that is used to monitor the food supply and to identify problem dietary exposures. Foods which are typical of a much larger group of foods and their ingredients are collected from many locations, prepared to table-ready state then analyzed. The concentration data are then applied to food consumption data. In Australia these are applied to the food consumption amounts for similar food categories in the national nutrition surveys [38] whereas in other countries, concentrations might be applied to a theoretical diet based on national survey data [39]. While this approach has its limitations, it allows a large number of analyses and an assessment of where there might be problems in dietary exposure or nutrient intake—either high for all chemicals, or low for nutrients—and directs where future work should be focused. 

## 4. Some Specific Considerations in Dietary Modeling

The formula to derive food chemical dietary exposures is the same as that used in research nutritional epidemiology: 

                  Dietary exposure = Σ(food chemical concentration × food consumption)                (1)

There are three different ways of operationalizing this formula. The first approach, the deterministic model, uses two single datapoints for each food—one for the population food consumption amount and one for the concentration of the chemical in the food of interest. For example, per capita disappearance data from national food balance sheets will permit an average dietary exposure or nutrient intake to be calculated but the population distribution in exposures/intakes cannot be estimated. This type of food “consumption” data may be all that is available in some countries. A common rule of thumb is to multiply the average dietary exposure estimate by three to generate an estimate of the extreme or high consumer dietary exposure or intake [40] and to compare these values to the reference health standards.

The second approach, semi-probabilistic (also called semi-distributional), is commonly used when analyzing food consumption data from a national nutrition survey, cohort or case-control study regardless of whether it is collected using a 24-h recall, record or food frequency questionnaire. Each food or food type described is given its own, but single, concentration value which is applied to food consumption data from many individuals and so a population distribution of exposure/intake of the food chemical/nutrient can be derived. The main question is which concentration to use: the mean, median, mode, maximum or other value? The answer depends on the purpose of the dietary modeling exercise and the data available.

The third, a probabilistic (or distributional) approach, uses the distribution of concentrations, rather than a single value, for each food, assuming such data are available. Thus there is a distribution for the chemical in the food(s) of interest and a distribution of the related food consumption amounts. A Monte Carlo approach is used to apply the food concentration data to the food consumption data for each individual to generate a population distribution. (This is based on the same underlying principles as the Probability Approach for estimating the proportion with inadequate nutrient intakes). 

The basic approach to modeling for food regulation purposes is to estimate dietary exposure now (at baseline) then project what it would be if the food regulation were to be amended. This involves running a series of exposure estimates using different proposed chemical concentrations in one or more foods, projecting the population dietary exposures and comparing them to the relevant health reference standard. More than one future scenario might be considered. For example, when selecting food vehicles for mandatory fortification, the concentration would decrease as the number of vehicles fortified increased. Depending on the food consumption patterns in different age-sex groups of the population, many different models might have to be run to identify the combination of concentration and vehicle/s that give the best combination of reach and increased nutrient intake in the target group but limits excess intake in other population groups. 

Because the goal of analysis is to clarify the impact of various possible regulatory decisions, it is not always necessary to develop a detailed dietary model. If a rough estimate, which can be done quickly with a deterministic model, clearly shows that there is no exceedance of the relevant reference health standard, then it might not be possible to justify the time required to assemble the detailed data to describe exactly how low the dietary exposures in the population are. However, when the quick method finds an exceedance, then further work is needed to remove the known overestimations and refine the estimate. 

### Estimating Usual (Long-Term) Intake in Semi-Probabilistic and Probabilistic Models

Several decades ago, national surveys in many countries ascertained only one day of food consumption information from each participant. Later, surveys have started to collect a second day of information from a subset of participants in some national surveys. Using analysis of variance, the data from the subset allows estimation of the within-person variance in nutrient intakes and, from this, a correction factor (s_b_/s_obs_), (the ratio of the standard deviation (SD) to the total SD) that can be used in the following formula [36]:

       Corrected value for a person = [(person’s value − group mean) × (s_b_/s_obs_)] + group mean    (2)

**Figure 4:**
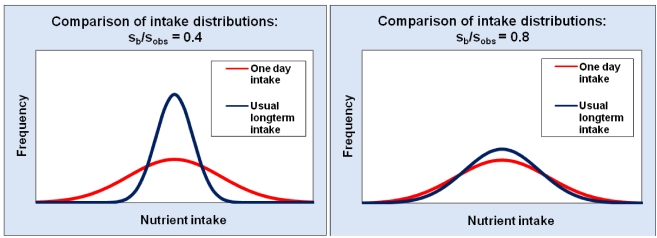
Illustration of the impact of two different between/total ratios (s_b_/s_obs_) in reducing the spread of an intake distribution estimated from collecting one-day of information from each survey participant to estimate the population distribution of long-term intakes of the same nutrient.

The corrected values are used to generate a more accurate estimate of the usual population distribution than is given by the one-day data. The impact of this correction can be substantial. For example, in the 1995 Australian National Nutrition Survey, the correction factor was 0.4 for zinc intakes in women aged 65 years and older [41]. In other words, the corrected SD of the zinc intake distribution (which estimates the usual long term intake of zinc) was only 40% of the SD of the one-day intake distribution (Figure 4). Consequently the range of long-term zinc intakes lying between −2SD to +2SD is less than half the width of the −2SD to +2SD range of the single-day distribution. Using the single-day distribution would greatly overestimate the proportion with high or low long-term zinc intakes although the direction of the error depends on whether the mean is above or below the health reference standard. By contrast, the correction factor was 0.8 for calcium for the same group [41]. Even so, the −2SD to +2SD range of long-term intakes is about three-quarters of the −2SD to +2SD range of the single-day intakes (Figure 4). 

This formula can be used with any distribution that can be approximately normalized [36] as would be the case for many nutrients. However, if the “usual” consumption amount of a particular food is of interest, for example apples, then there will be many people who did not eat apples on the survey day/s. Likewise, many people in a population would have no dietary exposure to a range of additives or other food components, depending on their food choices. In these cases, the distributions are not just skewed but can have a large peak at zero. Estimating long-term dietary exposures with this type of data is problematic. One option to avoid overestimation of the proportion of the population with high dietary exposures is to select a lower point on the exposure distribution (for example the 90th centile) as the point to compare to the reference health standard. A more expensive option is to collect multiple days of food consumption data from all survey participants and calculate average food consumption for each person. This approach is becoming more common in national nutrition surveys because it makes the data more useful for a range of users, including food regulators. Current research is investigating other, mathematical modeling solutions to this problem, although multiple days of intake data are still required [42,43,44]. 

## 5. Presentation of Results

Basic descriptive data, such as the mean and high dietary exposure values such as the 95th centile value (or low intakes for a nutrient adequacy assessment, 5th centile) should be presented for the total population and relevant subgroups. The proportion exceeding the reference health standard (or with inadequate nutrient intake) should also be given for the same groups. The level of exposure in relation to the reference health standard is useful when all dietary exposures are below the reference health standard (for example that the 95th centile of exposure is 1/10 of the ADI). A reference health standard is not an all-or-nothing cutpoint but one point on an underlying distribution. Consequently, different agencies may have their own policies about what results they report, particularly for high consumers. For example, will the 90th or 95th centile be reported? This will depend on the data and methods used for the dietary exposure assessment and agency policy. If single 24-h recall of food consumption data are used for the assessment, a 90th centile may be more appropriate because failing to account for within-person variation is likely to yield a wider distribution of exposures (Figure 4). When adjustments have taken place to predict usual exposures, the 95th centile could be used. With probabilistic dietary exposure assessments, a decision has to be made about what centile of exposure represents the high consumer; is it the 95th, 97.5th, 99th or 99.9th? The results may contain both consumers and non-consumers of the products that contain the chemical in question. A useful additional analysis is to repeat the analysis including only those who consume relevant foods containing the chemical. There may be other subdivisions that are relevant for some assessments, such as socio-economic status or region. 

## 6. Risk Management

Risk Managers (Figure 1) interpret any exceedance of a reference health standard in the light of the extent and severity of the adverse effects. This interpretation needs to be tempered by considering the quality of the data and assumptions that were made, whether there are important non-food sources of exposure that have not been considered, which population groups are most effected, the toxicological data used in the hazard characterization, *etc.* The decision is not made simply on the basis of a single figure. We further note that the reference health standards are based on the best data available at the time and the references for excess include the use of safety factors. Additional research for any chemical may lead to a revision of any reference, including the nutrient adequacy references, either up or down. 

### Maximum Limits for Food Chemicals in Food Regulations

One risk management approach is to set limits on the amount of chemical that might be permitted in foods or ingredients. We alert the reader to these (Table 3) because their names and abbreviations are somewhat similar to those of the reference health standards (Table 2) but they are not human health reference standards. They describe the maximum amount of certain chemicals that are permitted in food commodities, as set in food regulations, and are expressed per kg of the food. Enforcement agencies would compare the concentration of a chemical in the commodity (e.g., milk) to the MRL, *etc.* for that commodity directly to determine whether the food regulation is being complied with. On occasion, actual concentrations may be unknown and MRL, ML, *etc.* might be used as a surrogate for concentration in a particular food when calculating a full dietary exposure assessment.

**Table 3:** Nomenclature used in food regulations to describe maximum levels of food chemicals in foods set in food standards, used for assessing compliance (all expressed per kg food).

Food Chemical	Terminology	Abbreviation
Naturally occurring toxicants	Maximum Level	ML
Agricultural and Veterinary chemical residues	Maximum Residue Limit	MRL
Additives	Maximum Permitted Level	MPL
Contaminants	Maximum Level	ML

## 7. Risk Communication

The final stage of the Risk Analysis Framework considers Risk Communication (Figure 1). Risk Communication is the interactive process of exchange of information and opinion on risk among risk assessors, risk managers and other interested stakeholders [21]. Effective Risk Communication should address: the nature of the food risk; associated uncertainties and limitations (including those identified in the dietary modeling); risk management options; and how the selected risk management option addresses the risk. Communicating food regulation should be timely, meaningful, accurate and relevant to interested and affected audiences and be presented in a clear and understandable manner [5]. As noted earlier this communication needs to occur throughout the risk analysis process. 

## 8. Case Studies from Australia and New Zealand

### 8.1. Folic Acid Fortification [45]

Folic acid is not found naturally in food to any appreciable extent and the UL is for folic acid, not folate from any source. In Australia, voluntary fortification of selected types of food products had been permitted in 1995. Dietary modeling to project the impact of various options for mandatory fortification required an assessment of levels of folic acid in food based on prior permissions in the regulations (and discussion with manufacturers about practices). Only a limited range of foods needed to be included in the analysis because food composition data for natural folate were not relevant. Different scenarios were modeled, with correction to estimate long-term intake to determine what concentration of folic acid in which foods might lead to the greatest increase in folic acid intake in the target group (women of reproductive age) given the intake of folic acid with respect to the UL in other population groups. This information, combined with food technological and other information, was used to select bread-making flour as the vehicle and determine the concentration of folic acid in the vehicle for the purposes of mandatory fortification. Although FSANZ used its custom built dietary modeling computer program for this work, other countries may be able to use the WHO IMAPP [28] for such work.

### 8.2. Iodine Fortification [46,47]

Unlike folic acid, iodine is found naturally in food and so dietary modeling for mandatory iodine fortification included this as part of the baseline assessment. There had been no appreciable uptake of the voluntary permission to use iodized salt to manufacture food and so this did not need to be included in the baseline assessment although some domestic use of iodized salt was included. 

Salt was the only feasible carrier to fortify food with iodine identified by FSANZ. However, food composition tables describe total sodium levels in food rather than salt levels. Using the sodium concentration as a surrogate for salt content might have overestimated the amount of iodine that could be introduced because it would overestimate salt consumption. For example a large proportion of the sodium in some products using sodium bicarbonate as the raising agent might not be derived from salt and sodium propionate is one of the preservatives permitted in bread. Therefore FSANZ estimated the salt content of processed food based on the sodium content of each food but excluding sodium present naturally or derived from non-salt additives. The impact and reach of different fortification options was modeled using different concentrations of iodine in the salt in different combinations of food vehicles, with correction to estimate long-term intake, to determine what combination might lead to the greatest increase in iodine intake in the target group (women of reproductive age) given the intake of iodine with respect to the UL in other population groups. These analyses led to a decision to mandate the addition of iodine to salt used in making bread, using a defined concentration of iodine in the salt. Again, FSANZ used its custom built dietary modeling computer program but other countries may be able to use the WHO IMAPP [28] for such work.

### 8.3. Ferric Sodium EDTA [48]

An application was received to change the food regulations and add ferric sodium edetate (FeNaEDTA) to the list of iron compounds that could be used for voluntary iron fortification in those foods already permitted to contain other forms of added iron, without changing the amount of iron that could be added. Therefore allowing this compound would not increase the amount of iron in the population’s diet but would increase the EDTA intake. To model the potential increase, the quantity of EDTA in the diet from other EDTA-containing additives in the diet had to be estimated for the population at baseline and from projected future use of FeNaEDTA. Only foods permitted to contain EDTA-containing additives or iron from voluntary fortification were included in the analysis and a single-day intake was used. However, a proportion of the population had intakes exceeding the ADI for EDTA. Therefore a reduced model which excluded some foods with voluntary iron fortification permissions (breakfast cereals and formulated supplementary foods for young children) was run and gave satisfactory results. As a result, FeNaEDTA was permitted as a form of iron for voluntary fortification except for breakfast cereal and formulated supplementary foods for young children. 

### 8.4. Erythrosine in Craft Food Colorings [49]

Previously, the red food coloring, erythrosine, was permitted only in preserved cherries in Australia and New Zealand. FSANZ received an application to extend this permission to craft supplies to color icing and frosting. Therefore baseline dietary modeling estimated dietary exposure to erythrosine from preserved cherries including glace cherries and those found in canned fruit salad and various fruit cakes. As a first pass, the modeling to project what might happen if the permission were granted assumed that all icing used in cakes, including commercial cakes, would be colored with erythrosine at a level far higher than proposed in the application, and this was added to exposure from the cherries. Even under these assumptions, only 10–30% of the population consumed any foods that might contain erythrosine on the day surveyed. Even among those who had the highest potential dietary exposure to erythrosine, exposure was below 50% of the ADI. These results were enough to assess safety and it was not necessary to attempt to estimate the proportion of home-made cakes with icing containing red coloring to derive a more accurate exposure estimate. Additional scenarios for exposure to erythrosine in craft food colorings, for example from domestic use to color milk drinks, were also considered. FSANZ’s risk assessment concluded that the use of erythrosine as a food coloring in food containing icing at the proposed levels, did not raise any public health and safety concerns and the permission was granted.

### 8.5. Cyanogenic Glycosides in Cassava Chips [20]

All the above case-studies used semi-probabilistic modeling to estimate population exposures, some estimated a long-term exposure to a food hazard and others used single-day information which would overestimate high exposures. Following identification of cyanogenic glycosides in some cassava chips (crisps), FSANZ commissioned food analysis that showed that there were low levels of these glycosides in most cassava chips. However only a small proportion contained high amounts, *i.e.*, the distribution was highly skewed to the right. Consequently, using an average concentration would be likely to severely overestimate the proportion with high exposure and to underestimate the risk for those consumers eating small amounts of chips with high concentration levels of cyanogenic glycosides. The reference health standard for these glycosides is an Acute Reference Dose because illness can occur within hours of consumption. Because cassava chips had only recently been introduced to the market place, the consumption of potato chips (crisps) described in the earlier national nutrition survey was used as a surrogate in the dietary modeling owing to their similar appearance and nature and patterns of consumption. A full probabilistic model was calculated using both the distribution of glycoside concentrations in the cassava chips and distribution of serving sizes from occasions of eating potato chips among consumers only, without correction for within-person variability. As a result, an ML was set for cyanogenic glycosides in ready-to-eat cassava chips.

### 8.6. International Modeling

The FAO and WHO have developed a series of “cluster” diets (previously called “regional” diets) derived from international food balance sheets. There are 13 different diets, where countries are clustered according to consumption of the main staple(s) [50]. At international meetings such as the JMPR [33], the cluster diets are used to estimate dietary exposure to pesticide residues and contaminants at the international level through a simple deterministic calculation for long term exposure estimates only.

## 9. Conclusions

Risk assessment for food regulation brings together a range of epidemiological data to make public health decisions. The cross-sectional data analyses for dietary modeling can be extensive and sophisticated and contribute to decisions about whether a change in a food regulation might be warranted. It is used to evaluate exposure across a wide range of food chemicals from nutrients to contaminants and deliberate adulterants. Like all methods it is dependent on the quality of the data used and the skill of operator in understanding the goals. Although it can only approximate (model) the true situation, this information is critical to projecting the likely impacts of changes in food regulations before they happen. 
